# Case report: Magnetic resonance imaging findings of patients with diffuse uterine leiomyomatosis

**DOI:** 10.3389/fonc.2024.1430531

**Published:** 2024-07-03

**Authors:** Zanhua Zhang, Xianhui Lin, Xue Wang, Fang He, Weiwei Cai, Xiaoyan Min, Fei Xiang

**Affiliations:** ^1^ Department of ICU, The Second Affiliated Hospital and Yuying Children’s Hospital of Wenzhou Medical University, Wenzhou, China; ^2^ Department of Pathology, The Second Affiliated Hospital and Yuying Children’s Hospital of Wenzhou Medical University, Wenzhou, China; ^3^ Department of Radiology, The Second Affiliated Hospital and Yuying Children’s Hospital of Wenzhou Medical University, Wenzhou, China

**Keywords:** diffuse uterine leiomyomatosis, magnetic resonance imaging, case report, preoperative diagnosis, differential diagnosis

## Abstract

**Background:**

Diffuse uterine leiomyomatosis (DUL) is a seldom-seen condition, with only a handful of cases of magnetic resonance imaging (MRI) findings documented. In clinical settings, it is often mistaken for multiple uterine leiomyomas due to a lack of adequate recognition of DUL.

**Objective:**

This study shows two instances of DUL, underscoring their MRI findings to improve preoperative diagnostic precision.

**Conclusion:**

For patients exhibiting multiple uterine leiomyomas with masses present in the parametrial and abdominal cavities, consideration should be given to diagnosing DUL with DPL. The discoveries outlined in this paper furnish insights that can assist in directing treatment choices.

## Introduction

Uterine tumors are categorized as either benign or malignant. The predominant benign type is uterine leiomyoma (ULM), whereas endometrial cancer and uterine sarcoma are the most frequently observed malignant types. Uterine leiomyoma, also termed uterine fibroid, is a non-malignant, steroid-dependent tumor originating from the uterine muscle layer and represents the most prevalent benign tumor within the female reproductive system, occurring in approximately 20%–25% of women (1). This tumor is benign but commonly diagnosed, with ULM generally displaying slow growth rates. However, a rapid increase in size may indicate a potential for malignancy, such as sarcomas. Despite a limited response of the normal uterine muscle layer to estrogen, uterine fibroids demonstrate increased activity of estrogen regulatory genes and receptors and show an enhanced growth response to progesterone (2). It is noteworthy that although uterine leiomyomas are benign, there exists a risk of encountering an undiagnosed malignant tumor in one in 498 uterine tumors, such as leiomyosarcoma (3). Uterine leiomyoma and leiomyosarcoma share similar clinical and morphological features, making them challenging to differentiate; diagnosis is dependent on histological examination (4). Surgery remains the primary treatment option for ULMs and uterine leiomyosarcomas (ULMSs), thus underscoring the importance of precise preoperative diagnosis. Current diagnostic techniques, including ultrasound, CT, MRI, and CA-125 tests, fail to reliably differentiate between malignant and benign uterine fibroids. Therefore, a novel approach for differential diagnosis of ULM and ULMS is under investigation.

Diffuse uterine leiomyomatosis (DUL), classified as a borderline leiomyoma of the uterus, is distinguished by numerous small smooth muscle nodules that lead to a symmetrical enlargement of the uterus (5). These nodules vary in size, with the largest typically measuring up to 3 cm and most remaining under 1 cm in diameter (6). The smooth muscle cells of these nodules exhibit a uniform, bland, spindled shape with smaller dimensions compared to typical leiomyomas (7). DUL predominantly affects women of reproductive age and is a known cause of infertility (8). In clinical settings, DUL is often misidentified as multiple uterine leiomyomas due to its low recognition (9). Consequently, precise diagnosis of DUL is critical for appropriate treatment guidance. This report highlights two cases of DUL, focusing on the application of MRI to improve preoperative diagnostic accuracy.

## Case reports

### Patient 1

A 40-year-old unmarried woman with no children was admitted to the hospital, complaining of occasional abdominal bloating, fatigue, a history of uterine leiomyoma surgery 10 years ago, and abnormal menstruation for the past 5 years. Blood tests revealed severe anemia (hemoglobin level of 4.6 g/dL) and elevated serum tumor markers: a CA-125 level of 245.80 U/mL and a CA-199 level of 55.25 U/mL. Transvaginal ultrasound showed a notably enlarged uterus with multiple nodules displaying hypoechoic or uneven echoes. The uterine cavity line and endometrium were not visible, leading to the diagnosis of multiple uterine leiomyomas.

**Figure 1:**
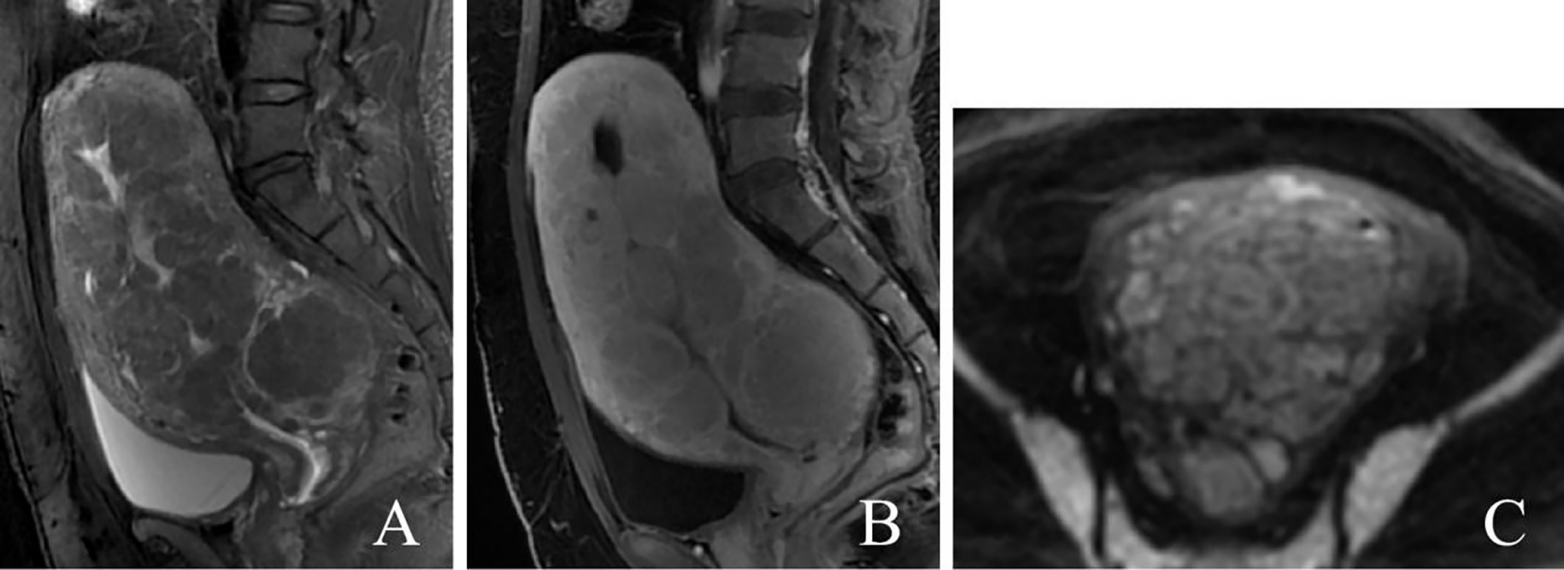
A 40-year-old patient with diffuse uterine leiomyomatosis. **(A–C)** Diffuse thickening of the myometrium with multiple masses showing a low signal in sagittal T2WI, mild enhancement in sagittal T1-enhanced image, and a slightly greater signal in DWI. T2WI, T2-weighted imaging; DWI, diffusion-weighted imaging.

MRI examination before surgery revealed that the uterine volume was significantly increased and that the myometrium showed multiple diffuse nodules and masses, which were isointense on T1-weighted imaging (T1WI) and had a slightly low signal on T2-weighted imaging (T2WI); additionally, some lesions showed heterogeneous signals and slightly high signals on diffusion-weighted imaging (DWI). The enhancement of the lesions was slightly lower than that of the uterine body (**Figure 1**). The diagnosis was multiple uterine leiomyomas. The right ovary showed a long cystic T1 and long T2 signal, measuring approximately 27 mm × 22 mm, with no obvious appearance on the enhanced scan.

The patient underwent a total abdominal hysterectomy, bilateral salpingectomy, right ovarian cystectomy, pelvic adhesiolysis, and pelvic uterine electrocautery. During the operation, the uterus appeared spherical, resembling that of a fifth-month pregnancy. A fibroid protrusion measuring approximately 6 cm × 6 cm × 7 cm was identified in the lower segment of the posterior uterine wall. Dense adhesions were observed between the anterior wall of the uterus and the bladder, as well as the lateral abdominal wall. Moreover, part of the mesentery was densely adhered to the posterior uterine wall. Multiple purple–blue nodules were noticed on the uterine surface and uterosacral ligaments. Additionally, the right ovary exhibited enlargement with a cyst measuring approximately 3 cm × 2 cm × 2 cm.

**Figure 2:**
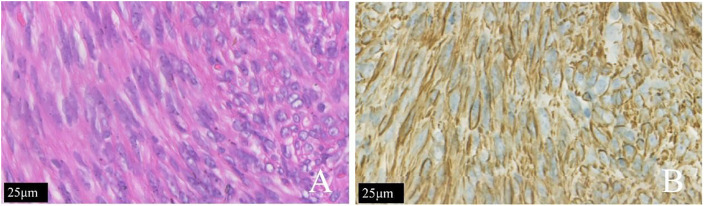
The same patient shown in **Figure 1**. **(A)** The tumor cells of the patient with diffuse uterine leiomyomatosis were long and fusiform. The cytoplasm was rich in red dye and was arranged in bundles or interleaved shapes according to H&E staining. **(B)** The SMA (+) is shown by immunohistochemical staining.

Histopathological examination of the hysterectomy specimen confirmed diffuse leiomyomatosis of the uterus and focally abundant cells with active mitotic activity (approximately 5–8/10 HPF). The immunostaining results were as follows: P53 (some positive), progesterone receptor (PR) (+), smooth muscle actin (SMA) (+), CD10 (−), Ki-67 (approximately 5%–10%+), and P16 (some +) (**Figure 2**).

### Patient 2

A 46-year-old married female, G2P2, was admitted to the hospital. The patient had a regular menstrual cycle, lasting 25–26 days, with a menstrual period lasting 6 days. There were no complaints of dysmenorrhea or alterations in vaginal discharge. However, 2 months ago, the patient reported a palpable lump in the lower abdomen, accompanied by intermittent lower abdominal swelling and pain. There were no associated symptoms such as fever, abnormal vaginal bleeding, nausea, vomiting, or discomfort such as frequent or urgent urination. The patient had previously undergone natural delivery and ligation. Additionally, 13 years ago, she had undergone open surgery for the removal of uterine fibroids.

Blood samples were collected, and the results were as follows: alpha-fetoprotein (AFP), 2.45 ng/mL; carcinoembryonic antigen (CEA), 0.83 ng/mL; CA-125, 103.20 U/mL; CA-199, 4.85 U/mL; and squamous cell carcinoma (SCC), 1.10 ng/mL.

An abdominal CT plain scan revealed large cystic and solid masses in the abdomen and pelvis, which were closely related to the bilateral adnexa. The volume of the uterus increased, and the bilateral adnexa was not clearly visible. CT enhancement was recommended.

**Figure 3:**
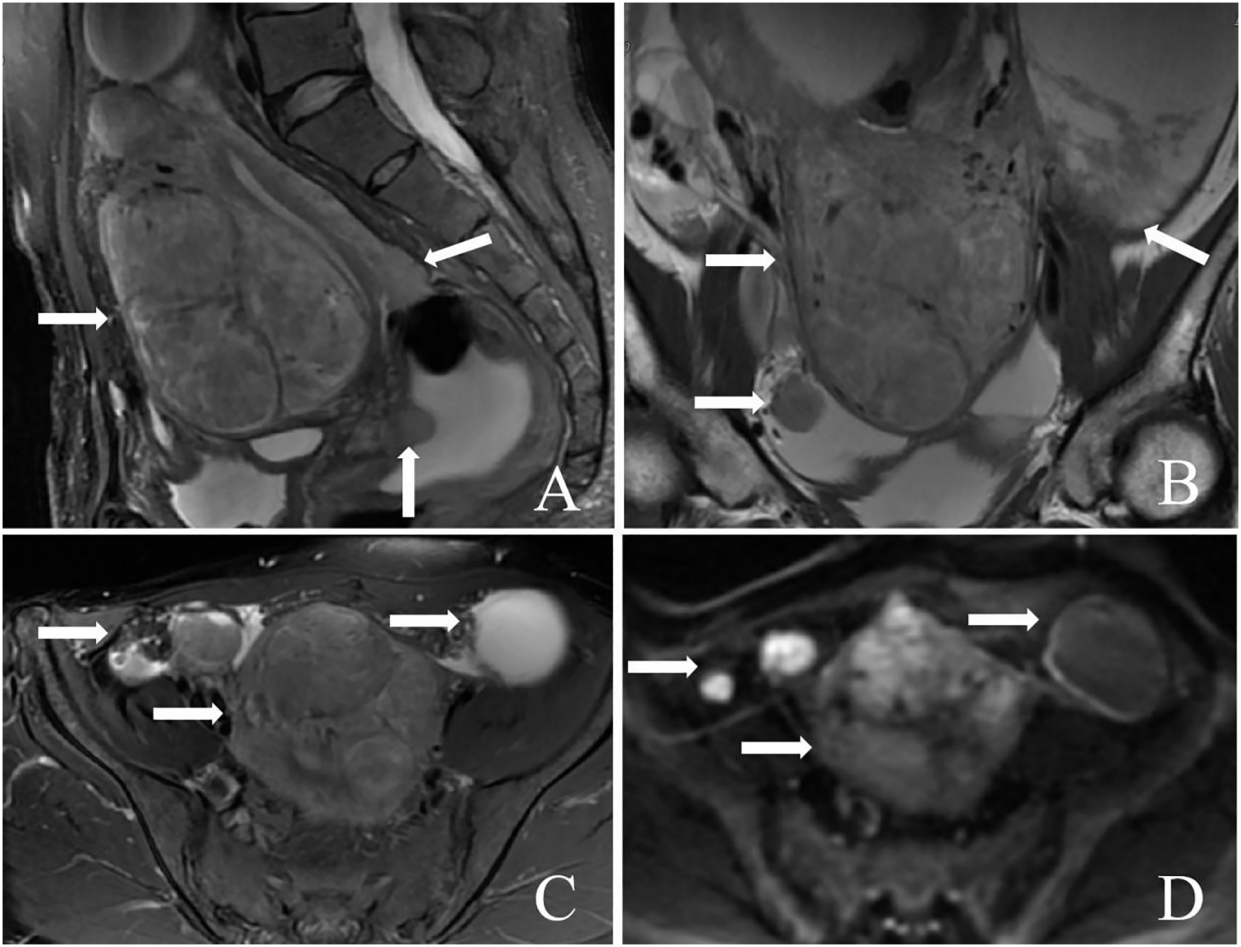
A 46-year-old patient with diffuse uterine leiomyomatosis (DUL) with peritoneal disseminated leiomyoma (DPL). **(A–D)** A large mass was observed in the anterior wall of the uterus. In addition, multiple masses were also observed in the posterior wall of the uterus and the abdominal and pelvic cavities beside the peritoneum (arrow). The masses above the myometrium and beside the peritoneum showed low signal intensity on sagittal T2WI, mild signal intensity on sagittal T1WI, and greater signal intensity on DWI. T2WI, T2-weighted imaging; T1WI, T1-weighted imaging; DWI, diffusion-weighted imaging.

Pelvic MRI revealed numerous cystic and solid masses present in the bilateral adnexal area, as well as in the parametrial and abdominal cavities. The largest mass, measuring 83 mm × 67 mm × 70 mm, was situated within the muscular layer of the anterior uterine wall. These masses displayed slightly hypointense signals on T2WI, isointensity on T1WI, and hyperintensity on DWI. The diagnosis indicated a typical uterine leiomyoma, while multiple lesions in the abdomen and pelvis were identified as metastatic tumors (**Figure 3**).

The patient underwent total hysterectomy, bilateral adnexectomy, and partial omentum resection while under general anesthesia. Several nodules, approximately 1.5 cm in diameter, were found attached to the greater omentum, positioned anterior to the uterus. These nodules were firm in texture and had smooth surfaces. Additionally, tumor protrusions measuring 10 cm and 12 cm were observed on the left and right sides of the uterine horn, respectively. These protrusions exhibited smooth surfaces, cystic degeneration, and multiple cysts and contained clear fluid. Ranging in size from 1.5 cm to 4 cm, the tumors had clear borders, smooth surfaces, and a non-thick cervix and showed no abnormalities in the appearance of the bilateral adnexa.

**Figure 4:**
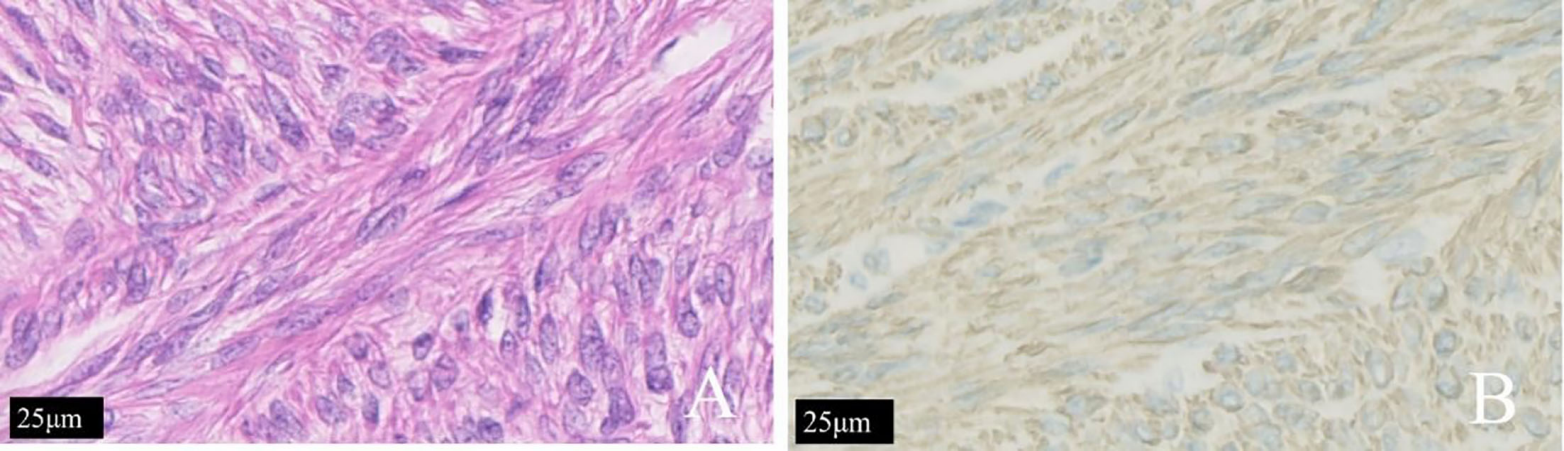
The same patient shown in **Figure 3**. **(A)** The tumor cells of patients with diffuse uterine leiomyomatosis were long and fusiform. The cytoplasm was rich in red dye and was arranged in bundles or interleaved shapes according to H&E staining. **(B)** Immunohistochemical staining for h-caldesmon (+) is shown.

Histopathological examination of the uterus, double adnexectomy, and nodules of multiple greater omentum specimens confirmed uterine diffuse leiomyomatosis with disseminated peritoneal leiomyomatosis (DPL), a hypercellular type. The immunostaining results were as follows: P53 (−), Ki-67 (<1% +), SMA (+), caldesmon (+), and CD117 (a few scattered +) (**Figure 4**).

## Discussion

The etiology of DUL remains uncertain, and unified diagnostic criteria have yet to be established. DUL predominantly impacts women of reproductive age (10), with instances reported in individuals as young as 16 years (11). The diagnosis is based on the imaging features observed through preoperative ultrasound or MRI. Patients with DUL show an enlarged uterus filled with numerous, poorly defined, small leiomyomas that replace much of the myometrium (12). In clinical settings, hysterectomy is the definitive treatment. Performing myomectomy on many nodules with unclear margins is challenging, and recurrence rates are high (7, 13). Furthermore, as DUL represents a borderline leiomyoma pattern, it warrants increased attention for its potential malignancy (14) compared to typical leiomyomas. Thus, accurate preoperative diagnosis of DUL is crucial.

Currently, no standard diagnostic approach for DUL exists. MRI facilitates early detection and precise preoperative assessment. Although ultrasound is recommended as the initial imaging technique for pelvic, uterine, or ovarian conditions, MRI is now the preferred and most effective method due to its multiplanar imaging capabilities, superior soft tissue contrast, and utility in pretreatment mapping. Imaging tests show diffuse leiomyomas with indistinct boundaries and merging, whereas typical leiomyomas appear as distinct masses with asymmetric uterine involvement. In two cases of DUL, the initial misdiagnoses included multiple ordinary leiomyomas and metastatic tumors. When MRI displays numerous, undefined leiomyomas replacing the majority of the myometrium, a diagnosis of DUL is possible. The second instance involved DUL with peritoneal disseminated leiomyoma, presenting multiple smooth muscles or smooth muscle-like nodules throughout the abdominal cavity, also known as leiomyomatosis peritonealis disseminata (15, 16).

Hysteroscopy, a standard surgical procedure for addressing intrauterine lesions, involves inserting a hysteroscope into the endometrial cavity via the cervical canal. A liquid expansion medium facilitates the observation and treatment of abnormalities within the uterine cavity. In the instances documented herein, both patients underwent total hysterectomy following a comprehensive evaluation of all relevant factors. It has been suggested that hysteroscopy may be a preferable option for patients with fetal DUL, as indicated by a case report of successful repeated pregnancies following “cold loop” hysteroscopic myomectomy (17). Additionally, successful pregnancies have been reported in women with DUL after undergoing hysteroscopic management (18). Conversely, recent findings in patients with incomplete spontaneous miscarriage have indicated that hysteroscopic surgery is not associated with higher subsequent delivery rates or greater safety compared to vacuum aspiration in those planning future pregnancies (19). Hence, further clinical data are required before definitive conclusions can be drawn. High-intensity focused ultrasound (HIFU) has been employed in patients with DUL, with studies indicating its safety, efficacy, and reliability. These findings support the use of drug therapy in conjunction with HIFU, yielding promising outcomes after conception (20).

Most cases of DUL with peritoneal disseminated leiomyoma recur following surgical intervention (21). Clinically, it is often initially misdiagnosed as peritoneal malignancy due to prominent imaging features, such as disseminated masses in the abdomen and pelvis. However, the absence of omental fatty infiltration, significant ascites, or solid organ metastases on imaging modalities like CT or MRI aids in distinguishing these DUL cases from malignant conditions (22). Additionally, well-circumscribed nodules are indicative of DUL with peritoneal dissemination. For the second patient documented in this report, the diagnosis was facilitated by the presence of a large uterine leiomyoma in the anterior wall of the uterus, exhibiting low signal intensity on T2WI with some hyperintensity due to hyaline degeneration. These characteristics, along with similar signal features of uterine leiomyoma in peritoneal masses, supported the diagnosis of DUL with peritoneal dissemination. Furthermore, most patients display normal blood levels of tumor markers, although CA-125 may be elevated. The features of well-circumscribed and similar signals of uterine leiomyoma for masses around the peritoneum in the abdomen and pelvis were helpful for the diagnosis of DUL with peritoneal dissemination. Moreover, most patients have normal blood levels of tumor markers, and CA-125 may be elevated. Finally, the results of histopathological examination can help to determine a definitive diagnosis, and the data can be positive for caldesmon, estrogen receptor (ER), PR, CD117, and muscle membrane antigen (SMA) (23).

Given the rare nature of DUL, only a few cases have been reported. Therefore, there are still many unknowns in the field. Moreover, different features might present in different patients. Therefore, more clinical data are needed to identify patient characteristics in MR images.

## Conclusion

In conclusion, DUL is a borderline leiomyoma with malignant potential and may be accompanied by peritoneal dissemination. Multiple uterine leiomyomas on MRI were innumerable and poorly defined in the myometrium, and DUL could be diagnosed. In addition, the bilateral adnexal area and the parametrial and abdominal cavities were noted. In this paper, our findings emphasized the importance of MRI in the accurate diagnosis of DUL. Given the rapid progress in artificial intelligence, it is reasonable to believe that algorithms can be developed to read MRI data with high efficiency and accuracy. For patients with multiple uterine leiomyomas with masses in the parametrial and abdominal cavities, a diagnosis of DUL with DPL should be considered. Finally, clinicians should be assisted in guiding treatment decisions.

## Data availability statement

The original contributions presented in the study are included in the article/supplementary material. Further inquiries can be directed to the corresponding author.

## Ethics statement

Ethical approval was not required for the studies on humans in accordance with the local legislation and institutional requirements because only commercially available established cell lines were used. Written informed consent was obtained from the individual(s) for the publication of any potentially identifiable images or data included in this article.

## Author contributions

ZZ: Writing – original draft. XL: Writing – review & editing, Conceptualization. XW: Writing – review & editing, Methodology, Supervision. FH: Writing – review & editing, Investigation. WC: Writing – review & editing, Project administration. XM: Writing – review & editing, Validation. FX: Writing – review & editing, Supervision.

## References

[1] Della Corte L Guarino MC Vitale SG Angioni S Mercorio A Bifulco G. C-section technique vs minilaparotomy after minimally invasive uterine surgery: a retrospective cohort study. Arch Gynecol Obstet. (2024) 309:219–26. doi: 10.1007/s00404-023-07239-7 PMC10769909 37796281

[2] Lippman SA Warner M Samuels S Olive D Vercellini P Eskenazi B. Uterine fibroids and gynecologic pain symptoms in a population-based study. Fertil Steril. (2003) 80:1488–94. doi: 10.1016/S0015-0282(03)02207-6 14667888

[3] Mas A Simón C. Molecular differential diagnosis of uterine leiomyomas and leiomyosarcomas. Biol Reprod. (2019) 101:1115–23. doi: 10.1093/biolre/ioy195 30184111

[4] Siedhoff MT Doll KM Clarke-Pearson DL Rutstein SE. Laparoscopic hysterectomy with morcellation vs abdominal hysterectomy for presumed fibroids: an updated decision analysis following the 2014 Food and Drug Administration safety communications. Am J Obstet Gynecol. (2017) 216:259.e251–259.e256. doi: 10.1016/j.ajog.2016.11.1039 PMC5835972 27890646

[5] Suminaga Y Taki M Okamoto H Kawamura Y Sagae Y Sunada M. A case of a patient with adhesive small bowel obstruction in pregnancy after extensive myomectomy for diffuse uterine leiomyomatosis. Case Rep Obstet Gynecol. (2022) 2022:3601945. doi: 10.1155/2022/3601945 36199388 PMC9529410

[6] Rosica G Santilli G Bucari D Amici B Bulletti F Patacchiola F. A case of disseminated peritoneal leiomyomatosis and diffuse uterine leiomyomatosis. Clin Exp Obstet Gynecol. (2011) 38:84–7. doi: 10.1111/j.1524-4741.2010.01035.x 21485735

[7] Konishi I. Diffuse leiomyomatosis: complete myomectomy for innumerable small nodules to achieve fertility sparing and childbearing. Surg J (N Y). (2020) 6:S50–s57. doi: 10.1055/s-0039-1693709 32399490 PMC7214083

[8] Lapan B Solomon L. Diffuse leiomyomatosis of the uterus precluding myomectomy. Obstet Gynecol. (1979) 53:82s–4s. 424137

[9] Silva CA Rosa F Rito M Cunha TM. Diffuse leiomyomatosis: A rare cause of a diffusely enlarged uterus. Radiol Case Rep. (2022) 17:1536–9. doi: 10.1016/j.radcr.2022.02.029 PMC8908024 35282327

[10] Purohit R Sharma JG Singh S. A case of diffuse uterine leiomyomatosis who had two successful pregnancies after medical management. Fertil Steril. (2011) 95:2434.e2435–2436. doi: 10.1016/j.fertnstert.2011.04.004 21549364

[11] Pai D Coletti MC Elkins M Ladino-Torres M Caoili E. Diffuse uterine leiomyomatosis in a child. Pediatr Radiol. (2012) 42:124–8. doi: 10.1007/s00247-011-2114-3 21710273

[12] Fedele L Bianchi S Zanconato G Carinelli S Berlanda N. Conservative treatment of diffuse uterine leiomyomatosis. Fertil Steril. (2004) 82:450–3. doi: 10.1016/j.fertnstert.2004.01.029 15302299

[13] Dai YX Feng FZ Leng JH Shi HH Cheng NH Wan XR. [Imaging features and clinical analysis of diffuse uterine leiomyomatosis cases]. Zhonghua Yi Xue Za Zhi. (2020) 100:2263–7. doi: 10.3760/cma.j.cn112137-20200307-00634 32746595

[14] Otsubo Y Nishida M Arai Y Ichikawa R Sakanaka M. Diffuse uterine leiomyomatosis in patient with successful pregnancy following new surgical management. Arch Gynecol Obstet. (2014) 290:815–8. doi: 10.1007/s00404-014-3309-2 24930118

[15] Willson JR Peale AR. Multiple peritoneal leiomyomas associated with a granulosa-cell tumor of the ovary. Am J Obstet Gynecol. (1952) 64:204–8. doi: 10.1016/S0002-9378(16)38757-9 14933538

[16] Thang NM Thien DH Huyen Anh NT Cuong TD. Leiomyomatosis peritonealis dissemianata five years after laparoscopic uterine myomectomy: A case report. Ann Med Surg (Lond). (2021) 66:102377. doi: 10.1016/j.amsu.2021.102377 34026111 PMC8131390

[17] Mazzon I Favilli A Grasso M Morricone D Di Renzo GC Gerli S. Is 'cold loop' hysteroscopic myomectomy a better option for reproduction in women with diffuse uterine leiomyomatosis? A case report of successful repeated pregnancies. J Obstet Gynaecol Res. (2015) 41:474–7. doi: 10.1111/jog.12548 25330711

[18] Zhao H Yang B Li H Xu Y Feng L. Successful pregnancies in women with diffuse uterine leiomyomatosis after hysteroscopic management using the hysteroscopy endo operative system. J Minim Invasive Gynecol. (2019) 26:960–7. doi: 10.1016/j.jmig.2018.10.003 30308306

[19] Huchon C Drioueche H Koskas M Agostini A Bauville E Bourdel N. Operative hysteroscopy vs vacuum aspiration for incomplete spontaneous abortion: A randomized clinical trial. Jama. (2023) 329:1197–205. doi: 10.1001/jama.2023.3415 PMC10091175 37039805

[20] Gong C Lin Z Deng Y Yang B Zhang L. Successful pregnancies in women with diffuse uterine leiomyomatosis after high-intensity focused ultrasound ablation: report of three cases. Int J Hyperthermia. (2023) 40:2234674. doi: 10.1080/02656736.2023.2234674 37437896

[21] Żyła MM Dzieniecka M Kostrzewa M Stetkiewicz T Wilamowska A Księżakowska-Łakoma K. Leiomyomatosis peritonealis disseminata of unusual course with Malignant transformation: case report. Acta Obstet Gynecol Scand. (2015) 94:220–3. doi: 10.1111/aogs.12549 25546607

[22] Belmarez JA Latifi HR Zhang W Matthews CM. Simultaneously occurring disseminated peritoneal leiomyomatosis and multiple extrauterine adenomyomas following hysterectomy. Proc (Bayl Univ Med Cent). (2019) 32:126–8. doi: 10.1080/08998280.2018.1520555 PMC6442858 30956607

[23] Chen X Liu H Shi H Fan Q Sun D Lang J. Leiomyomatosis peritonealis disseminata following laparoscopic surgery with uncontained morcellation: 13 cases from one institution. Front Surg. (2021) 8:788749. doi: 10.3389/fsurg.2021.788749 34957207 PMC8695543

